# Flipped Well-Plate Hanging-Drop Technique for Growing Three-Dimensional Tumors

**DOI:** 10.3389/fbioe.2022.898699

**Published:** 2022-07-04

**Authors:** Yoon Jeong, Ashley Tin, Joseph Irudayaraj

**Affiliations:** ^1^ Department of Bioengineering, University of Illinois at Urbana‐Champaign, Urbana, IL, United States; ^2^ Cancer Center at Illinois, University of Illinois at Urbana-Champaign, Urbana, IL, United States; ^3^ Department of Computer Science, University of Illinois at Urbana‐Champaign, Urbana, IL, United States; ^4^ Carl R. Woese Institute for Genomic Biology, University of Illinois at Urbana-Champaign, Urbana, IL, United States

**Keywords:** flip well-plate, hanging drop, tumor spheroid, scaffold-based, 3D culture

## Abstract

Three-dimensional (3D) tumor culture techniques are gaining popularity as *in vitro* models of tumoral tissue analogues. Despite the widespread interest, need, and present-day effort, most of the 3D tumor culturing methodologies have not gone beyond the inventors’ laboratories. This, in turn, limits their applicability and standardization. In this study, we introduce a straightforward and user-friendly approach based on standard 96-well plates with basic amenities for growing 3D tumors in a scaffold-free/scaffold-based format. Hanging drop preparation can be easily employed by flipping a universal 96-well plate. The droplets of the medium generated by the well-plate flip (WPF) method can be easily modified to address various mechanisms and processes in cell biology, including cancer. To demonstrate the applicability and practicality of the conceived approach, we utilized human colorectal carcinoma cells (HCT116) to first show the generation of large scaffold-free 3D tumor spheroids over 1.5 mm in diameter in single-well plates. As a proof-of-concept, we also demonstrate matrix-assisted tumor culture techniques in advancing the broader use of 3D culture systems. The conceptualized WPF approach can be adapted for a range of applications in both basic and applied biological/engineering research.

## Introduction

The practical utilization of a 96-well plate for biological inquiry enables the execution of parallel and large-scale independent experiments. Harnessing the benefits of the standard 96-well plate format, 3D spheroidal cultures with compatible designs [*e.g.*, handing drop insertion (Tung et al., 2011; Zhao et al., 2019; Fu et al., 2021; Liu et al., 2021), including magnetic levitation (Kim et al., 2013; Tseng et al., 2015; Ge et al., 2018), and nonadhesive surface (Friedrich et al., 2009; Liao et al., 2019)] were developed to address several applications. Products such as the ultra-low attachment (ULA) plates are available (Vinci et al., 2012; Raghavan et al., 2016) along with other techniques for 3D spheroidal culture development, for example, microfabrication (Lee J. M et al., 2018; Park et al., 2018; Ganguli et al., 2021; Sun et al., 2021), soft lithography (Cha et al., 2017; Kuo et al., 2017), and floating methods (Vadivelu et al., 2015; Kim et al., 2021) among others. Despite the widespread interest, the current culture methods have several limitations, including nonuniformity of spheroid formation, irregular sphericity, short-term cultures, evaporative loss of culture media, and limited volume (size of spheroids) of culture (Shi et al., 2018; De Souza Moraes et al., 2020; Han et al., 2021). Furthermore, many of the existing methods are expensive, labor-intensive, and highly complex, limiting their applicability and standardization. Furthermore, most of the 3D spheroid culturing methodologies have not progressed beyond the inventors’ laboratories. A simple and user-friendly approach for 3D spheroid production, especially long-term growth, will render the widespread utilization of the spheroid technology for more meaningful biological evaluation beyond what 2D cultures can afford.

**FIGURE 1:**
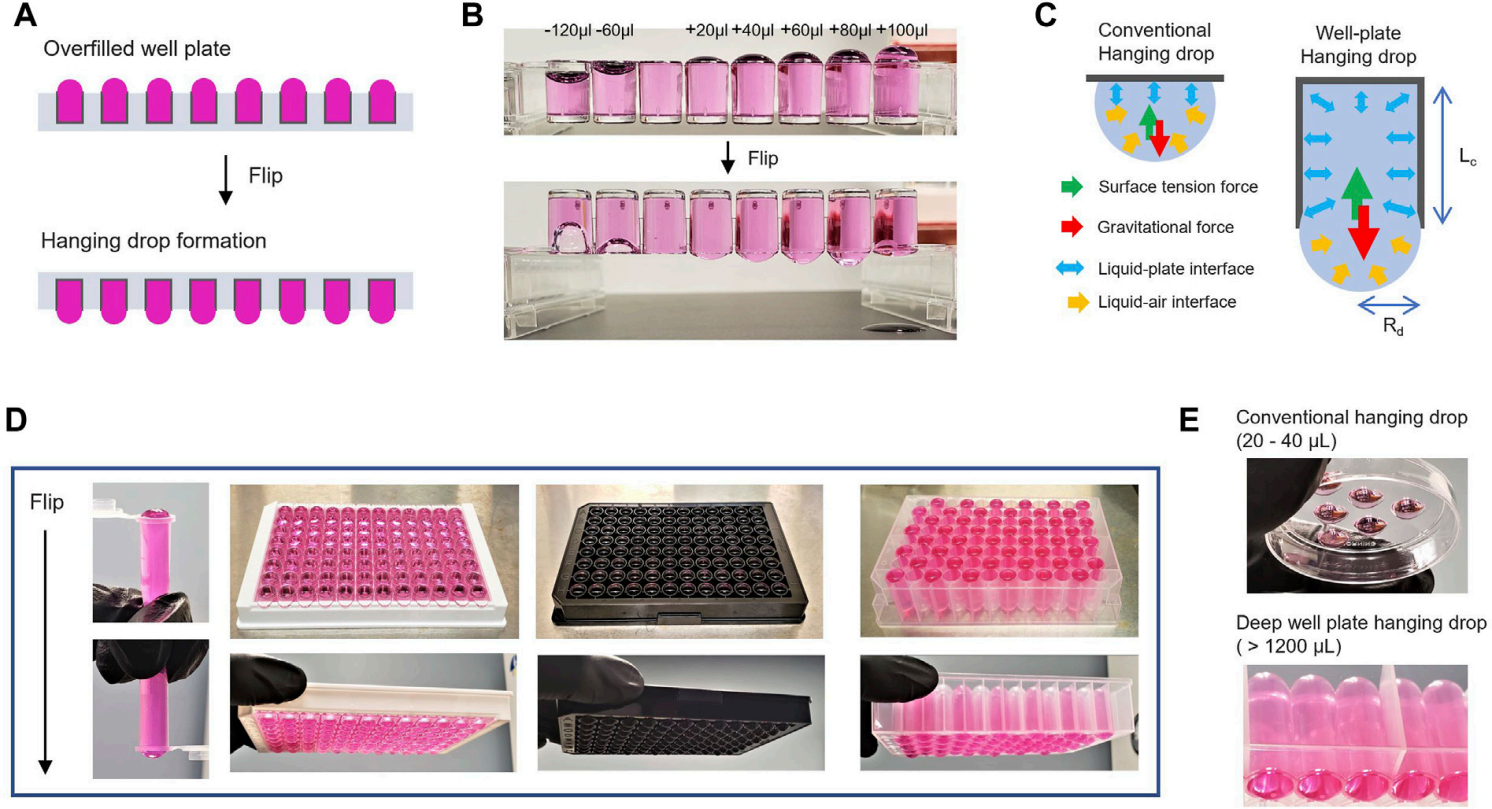
Hanging drop formation by well-plate flip. **(A)**. Schematic illustration of an overfilled and flipped well-plate to generate hanging drop meniscus. **(B)**. Images of 96-well-based strips to compare underfilled wells (−120 and −60 μl) and overfilled wells (20–100 μl) to the maximum volume of a well before/after flipping the plate. **(C)**. Comparison of conventional hanging drop and well-plated hanging drop formation. The mechanistic rationale of hanging drop formation generated at the bottom of a flipped well-plate. R_d_ is the radius of the pendant drops, and L_c_ is the capillary length of the well-plate. **(D)** Representative images of the flipped tube and well-plate overfilled with 60 μl from the maximum volume of each designed dimension (from left to right: 0.7 ml microcentrifuge tubes, Elisa 96-well strip, Black 96-well plate, and 1.1 ml Deep 96-well plate). **(E)** Images of conventional hanging drop in the lid of a plastic plate and deep well-plate hanging drop.

Our study aims to introduce a 3D tumor spheroid technology utilizing a matrix-assisted culture approach in a standard 96-well plate format. By simply flipping a universal 96-well plate, pendant drop meniscus can be formed in single wells of the plates where large spheroids can be generated in a contact-free environment (Figure 1). Homogenous 3D spheroids that can be maintained for a prolonged time could give rise to the formation of tumoral physiological features observed in cancers *in vivo* (Costa et al., 2016; Nath and Devi, 2016; Decarli et al., 2021). However, the realization of such a technology has remained a technical challenge. To demonstrate the broad applicability of the flipped 96-well system, we first show the generation of large scaffold-free 3D tumor spheroids in millimeter scale (over 1.5 mm in diameter), without any structural support. The self-assembled spheroid models can be cultured over a month and possibly longer, with high morphological homogeneity and excellent sphericity.

The hanging drop technique has primarily been used as a scaffold-free technique to develop cancer spheroids (Decarli et al., 2021). The hanging drop meniscus in contact-free format is significantly advantageous because other scaffold-based 3D culture approaches can be incorporated with a large working volume of the culture media. In conventional hanging drop studies, the long-term maintenance of tumor culture models in scaffolds has not been possible primarily due to the small working volume of the culture media (20–40 μL of hanging drop) (Foty, 2011; Tung et al., 2011). We propose a flipped well plate concept to generate a contact-free environment to provide sufficient working volume (over 1 ml per well) for cultures to develop without modification. To demonstrate the significance, we adopted an *in situ* 3D culture technique with matrix-assisted design [i.e., synthetic or natural scaffold (Li and Kumacheva, 2018)] that promotes the interaction between the cells and the extracellular matrix (ECM) environment. By extending the concept of 3D matrix culture in hanging drop format, natural tissue-derived scaffold-based 3D tumor cultures in physiologically relevant conditions were demonstrated. The conceptualized well-plate flip (WPF) approach poses no methodological barrier in translation and can be adapted for a range of applications in both basic and applied biosciences research.

## Materials and Methods

### Cell Culture

Human colorectal carcinoma cells (HCT116), obtained from American Type Culture Collection (ATCC), were maintained in Dulbecco’s Modified Eagle Medium (DMEM; Corning), supplemented with 10% fetal bovine serum (FBS; Gibco) and 1% penicillin/streptomycin (PS; Lonza). Other malignant cells (A375, skin melanoma; HepG2, hepatocellular carcinoma; Panc-1, pancreatic carcinoma; A498, renal carcinoma; and Du145, prostate carcinoma) and human lung fibroblast (IMR-90 fibroblast, ATCC-CCL-186) were obtained from the Tumor Engineering and Phenotyping Facility of the Cancer Center at Illinois (CCIL, University of Illinois Urbana-Champaign). All the cells were cultured and maintained in a humidified 5% CO_2_ incubator (Thermo Scientific) at 37 °C, according to routine standard protocols unless otherwise noted for culture conditions.

### Humidity Control Chamber

A prototype chamber (Supplementary Figure S1) was designed for humidity maintenance and printed at Illinois MakerLab (http://makerlab.illinois.edu). A bench-top 3D printer (Ultimaker) was used to fabricate a humidity control chamber using polylactic acid (PLA) filament, designed by Sketchup software (Trimble). All of the standard 96-well plates (Corning, Thermo Scientific, CytoOne, Advangene, and Genesee Scientific), Elisa 96-well strip (Corning), deep 96-well plate (Effendorf), and black 96-well plate (Greiner Bio) were tested to generate hanging drop formation and evaluated for the evaporation rates of hanging drop height in the chamber. Variation in evaporation from the height of each hanging drop was measured every 6 h using standard 96-well plates flipped in the chamber, which was maintained in a humidified incubator at 37°C in 5% CO_2_. A standard 96-well plate (Thermo Scientific) was mainly utilized without modification or treatment in this study unless otherwise noted for specific types of well plates.

### Spheroid Generation

HCT116 (human colorectal carcinoma cells) 3D spheroids were generated using a standard 96-well plate at the bottom of the hanging drop meniscus formed by the well-plate flip technique. The seeding density of cells varied from 2 × 10^4^ to 3 × 10^2^ cells per well before the 96-well plate overfilled with 440 μl volume of media flipped to produce a hanging drop meniscus. For long-term incubation of over 1 month, culture media was repeatedly replenished by either manual liquid pipetting or a well–well transfer technique (Supplementary Figure S4). 3D spheroids were monitored daily using an inverted microscope (Leica DMI3000B) equipped with a CCD camera (Qimaging EXi Blue). The size distribution of the tumor spheroid was analyzed from the optical microscopy images by open-source imaging software (Fiji ImageJ). (Schindelin et al., 2012; Chen et al., 2014).

### WST-1 Proliferation Assay and Supernatant ATP Assay

Spheroid proliferation incubated for 5 days was measured through the WST-1 assay (Roche Applied Science), and then after 48 h, other groups of spheroids were measured to compare drug sensitivity to 0.5 μM of 5-Azacytidine (Sigma). For ATP determination in culture media, 150 μl of supernatant of spheroid culture was collected at each time point. The ATP contents in the supernatant were evaluated using a luciferase-based assay (Mahmud et al., 2009). Briefly, the supernatant collected was mixed with firefly lantern extracts (Sigma/FLE-250) and incubated in D-luciferin solution (GoldBio/LUCK-100) for 10 min at 37°C. To detect the luciferase activity, a Synergy H1 multimode microplate reader (Biotek) was used by comparing to an ATP standard curve (Sigma/A2383). Relative ATP concentrations were calculated as the difference in ATP level compared with the standard dilutions curve. Data were expressed as a relative fold of ATP levels.

### Drug Discrepancy Test in 2D and 3D Culture

For 2D cultures, HCT116 cells were seeded on standard 96-well plates at a density of 5 × 10^3^ per well and preincubated for 12 h. 5-Azatididine (Sigma) and docetaxel (Sigma) were used to evaluate the cytotoxicity of the anticancer drug. The cells were subsequently incubated in various concentrations of the drugs for 24 h, and then WST-1 (Roche Applied Science) assay for cytotoxicity was performed to determine the 50% inhibition concentration (IC50) value. Sigmoidal dose–response curves for 2D culture were generated using Origin 2019 software (Origin Lab), and the IC50 value was obtained. The IC50 value of the drugs was used to verify a drug discrepancy in 3D cultures at each drug concentration.

The assessment of drug-dose response in 3D spheroids was performed according to the standard colony-forming assay (CFA) (Franken et al., 2006). For 3D cultures, tumor spheroids were prepared as described above using the well-plate flip technique in a standard 96-well plate with 2 × 10^3^ cells per well in 440 μl of culture medium. Large tumor spheroid prepared for 3 weeks was dissociated by trypsinization to determine reproductive cell death after drug treatment for 24 h with the IC50 concentration of 5-azatididine and docetaxel. The dissociated cells were seeded at various densities on 12-well plates and allowed to grow for 10 days per established protocols. ImageJ software customized with Fiji macros was used to calculate the number of colonies formed on the plates. The results of drug-dose resistance represent the cell death ratio of the IC_50_ cultured in 2D and 3D culture systems and compared with a negative control group (nondrug treatment).

### Flow Cytometry

Flow cytometric analyses of 3D spheroid samples were performed for Live/Dead assay using Calcein-AM (Invitrogen) and Propidium Iodide (Invitrogen). For 3D recovery, tumor spheroids of different sizes were harvested and re-suspended for spheroid dissociation enzymatically through trypsinization with 0.25% trypsin-EDTA for 5 min at 37°C. The dissociated cells were washed twice in PBS and incubated with fresh culture media containing 1 μM of Calcein-AM at 37°C for 30 min. The cells were washed twice with PBS and stained using Propidium Iodine at final concentration of 1 μg/ml. Samples were subsequently assayed with a BD LSR II flow cytometer (BD Biosciences) after cell density was adjusted according to the requirements of the flow cryometric analysis. Data were collected with the FACSDiva 6.1.1. software (BD Biosciences). FCS Express 6 software (De Novo) was used to analyze the data.

### Glucose Consumption and Lactate Production

Standard enzyme reagent kits were used to measure relative glucose and lactate concentrations. Spheroids generated in different conditions were obtained 3 and 7 days after seeding the HCT116 cells. Spheroid cultures were transferred and incubated in PBS for 2 h. Then the culture medium (glucose content of 4.5 g/L) was changed and incubated for an additional 12 h to measure relative glucose/lactate levels; 200 μl medium were taken from each sample and analyzed using the Glucose (GO) Assay Kit (Sigma/GAGO20-1 KT) and the colorimetric l-lactate assay kit (Sigma/MAK329-1 KT). The absorbance at 540 nm for the glucose assay and at 570 nm for the lactate assay was measured in a Synergy H1 multimode microplate reader (Biotek). Spheroid samples were lysed in 1% (m/v) sodium dodecyl sulfate (SDS, Sigma) solution in PBS, and protein concentration was determined with standard bicinchoninic acid (BCA) assay (Thermo Scientific). Glucose consumption and lactate production rates in spheroid cultures were determined and compared with total protein concentration.

### Migration Observation

HCT116 3D spheroids were generated by the flip well plate method described above and allowed to settle at the bottom of the well-plate for subsequent operations by flipping. Tumor spheroids generated for 3 days were placed onto the flat-bottom of the 96-well, and spheroid migration was observed for 72 h. Other spheroidal samples cultivated for 5 days were prepared with or without the final concentration (10 nM) of docetaxel for 24 h. Optical microscopic images of the spheroids are then captured every 12 h for a period of up to 48 h.

### Gel Matrix Embedment

After culturing for a period of up to 15 days, tumor spheroids generated at the bottom of the hanging drop meniscus settle to the U-shaped bottom of the 96-well plate by flipping. A single spheroid was placed at the bottom of the 96-well plate, and then the culture media was carefully discarded. Tumor spheroid of different sizes was embedded into either 0.5 wt% of agarose gel (Fisher BioReagents/BP160-100) or Matrigel membrane matrix (Corning/356234) and then cultured for up to 10 days. For gel matrix embedment, 10 μl of agar solution was slowly dispensed onto the surface of the well plate for 15 min at room temperature to solidify. Then, 50 μl of agar solution was slowly dispensed dropwise onto the plate to completely cover the spheroid in agar gel. After complete gelation for 15 min, 100 μl of fresh medium was added to the well plate. Similarly, 10 μl of Matrigel solution cooled at 4°C was slowly dispensed onto the surface of the well plate and preincubated for 30 min at 37°C to solidify. Then, 50 μl of Matrigel solution was dispensed on top of the spheroid to embed the spheroid completely in the Matrigel. The gelation of Matrigel was performed at 37°C overnight in a humidified incubator, and then 100 μl of media was added to the plate. Optical microscopic images of the spheroids embedded in the gel were then captured every day. An additional experiment of U87 spheroid embedded in the Matrigel is provided in Supplementary Figure S8.

### Co-culture with 2D Fibroblast and Dissemination

For co-culture and dissemination assay with 2D fibroblasts, IMR-90 human fibroblasts were seeded in a standard 96-well plate at two different densities. IMR-90 cells were precultured until 20% or 40% confluence on 2D plate. Tumor spheroids generated separately for 10 days were transferred into the 96-well plate with different confluency of IMR-90 fibroblast. For co-culture studies, the 96-well plate was overfilled up to 440 μl volume of media and then flipped to produce hanging drop meniscus. For 3D spheroid dissemination assays, tumor spheroids were mounted on top of a confluent fibroblasts layer for a desired time period after co-culture by flipping the well-plate again. Other tumor spheroids generated by the same approach were transferred onto the well plate in which 3D tumor spheroid and 2D fibroblasts were present. Optical microscopic images of tumor spheroids with IMG-90 fibroblasts were then captured daily.

### Hydrogel-Based Hanging Drop 3D Culture

A mixed hydrogel of alginate (0.5 wt%) and gelatin (1 wt%) was utilized as a 3D scaffold substrate. Briefly, 0.5 wt% of alginate solution and 1 wt% of gelatin solution were prepared by dissolving sodium alginate (Sigma/71238) and gelatin (Sigma/G9382) powders in PBS. Re-suspended HCT116 cells prepared with standard procedures were mixed with 1 ml of the gelatin/alginate solution at a density of 10^5^ cells/ml. Bead-type hydrogels were generated by adding the mixture solution with cells in a 0.1M CaCl_2_ (Sigma-Aldrich) solution in a dropwise manner. After gelation for 15 min, bead hydrogels containing HCT116 cells were washed twice in DI water and cultivated in a hanging drop format for over 1 month and the media was replenished (every 3 days for the first 2 weeks and every 1 day for the last 2 weeks manually).

### Decellularized Tissue-Based Hanging Drop 3D Culture

Colonic tissue was obtained from CD-1 male mice sacrificed under UIUC IACUC approved protocol and immediately frozen at −80°C. Prior to decellularization, mouse intestinal samples were washed three times in PBS. Decellularization was performed by immersion of the tissue in a cell lysis B-PER reagent (Thermo-fisher) for 48 h at room temperature, and the decellularized tissue was then washed three times in PBS. The fragmentation of residual DNA in decellularized samples was lysed using DNAse I (New England Biolabs) at a concentration of 10 U/ml (1 U is the enzyme activity) prepared in reaction buffer. The DNA contents of decellularized tissue samples were extracted by Purelink genomic DNA mini kit (Invitrogen) following the manufacturer’s instructions and quantified with NanoDrop One microspectrophotometer (ThermoFisher). For evaluation of recellularization, decellularized intestine and HCT116 cells prepared with standard protocols at a density of 10^4^ cells/well were incubated together for 1 month in hanging drop formation. Media were replenished manually every 2 days after 1 week of incubation.

### Histological Analyses

Slide sections were stained according to established protocols. Briefly, samples were fixed in 4% neutral buffered formalin (NBF; Sigma) solution at 4°C overnight and sequentially processed in a tissue processor (Leica Microsystems) as immersed with 70%–80% ethanol for 10 min each, 95% ethanol for 15 min, and 100% ethanol for 10 min. Next, the samples were placed in xylene solution for 10 min following standard protocols and immersed into three paraffin baths, 15 min in the first bath and 25 min in the other paraffin baths, and then placed in a mold with paraffin. Paraffin-embedded samples were cut into 5-μm-thick sections and mounted onto hydrophilic slide glass. After deparaffinization with xylene, samples were then hydrated by ethanol and distilled water. Samples were stained with hematoxylin and eosin (H&E; Sigma) and Periodic acid-Schiff (PAS; Sigma).

For immunohistochemistry experiments for Ki-67 (Invitrogen; PIMA514520, 1:200 dilution), deparaffinized sections were quenched by incubating the slides in 2.5% hydrogen peroxide to block endogenous peroxidase. Sections were permeabilized for 20 min in 0.1% (v/v) Triton X-100 in PBS. Sections were incubated with primary antibodies applied at 1:200 dilution in 0.1% (w/v) bovine serum albumin (BSA) in PBS overnight at 4°C. The immunocomplexes were stained using the avidin–biotin–peroxidase complex (ABC) method (Vector Laboratories). Hematoxylin counterstain was performed for the identification of cell nuclei. Ki-67 proliferation index was calculated as a percentage of stained nuclei from digitized images of the area with the highest number of brown nuclei, according to established protocols using ImageJ software (Mezei et al., 2011).

### Statistical Analysis

The data for the experiments were expressed as mean ± SD and analyzed by unpaired Student’s *t*-test and one-way ANOVA with a post hoc test (Graphpad prism 9.0.), where post hoc comparisons were conducted using the Tukey’s method. *p < 0.05, **p < 0.01, and ***p < 0.001, ****p < 0.0001 were considered as statistically significant. Details of the statistical tests are presented in each figure legend. No statistical analysis was performed to predetermine a required effect size.

## Results

### Pendant Drop Formation and Maintenance in Single Wells

In the WPF method depicted in Figure 1A, the adhesion force of the liquid media to polystyrene plastic in the well causes an upward capillary action against gravity (Figure 1B). This action resulted in a concave meniscus pulled into a spherical shape at the air–liquid interface. The formation of a pendant drop, first examined by Young and Laplace two centuries ago, has been well-understood. The relative conditions for surface gravitational force and surface tension as characterized by the *Bond* number (N_b_) (Wilkes et al., 1999; Berry et al., 2015) (Figure 1C) are given by,

$$N_{b}≃{\left(R_{d}/L_{c}\right)}^{2}$$

where N_b_ is the gravitational force/surface tension force (Lee et al., 2010), R_d_ is the radius of the pendant drops, and L_c_ is the capillary length (depth) of a well in the plate. When a 96-well plate overfilled to the maximum capacity in each well is inverted, a balance exists between the surface tension and gravitational force, which prevents the liquid media from collapsing and spilling the contents in the well. Interestingly, this principle can be applied to any standard experiment with the 96-well plate (Figure 1D).

In conventional hanging drop methods, the small working volume (20–40 μL) on the lid of a plastic plate or in different configuration settings has been a major impediment for culture growth because of the rapid evaporative loss of the small volume of the fluid (Figure 1E) (Tung et al., 2011; Klingelhutz et al., 2018; Zhao et al., 2019; Ganguli et al., 2021). In our conceptualized approach, it is possible to use a deep well-plate with up to 1 ml volume in each well to generate pendant drop meniscus, even with the increased capillary length (L_c_) of the well depth.

Traditionally, a semi-closed system sealed with a reservoir is employed to compensate for the loss due to evaporation in the hanging drops (Erbil and Dogan, 2000; Martins et al., 2008). For efficient maintenance, we designed a prototype chamber to examine the flip concept technique, which eliminates the laborious maintenance steps of hanging drop well-plates (Supplementary Figure S1). A plastic chamber designed with a 3D printer that can stack multiple standardized well-plates can be used as an effective control system for hanging drop maintenance in a humidified incubator. Further, we demonstrate a simple strategy for the utilization of a spacer that can be inserted between a flipped well-plate and a lid reservoir to maintain hanging drops. The conceptualized design is flexible and can be tailored for specific applications.

### 3D Spheroid Generation

**FIGURE 2:**
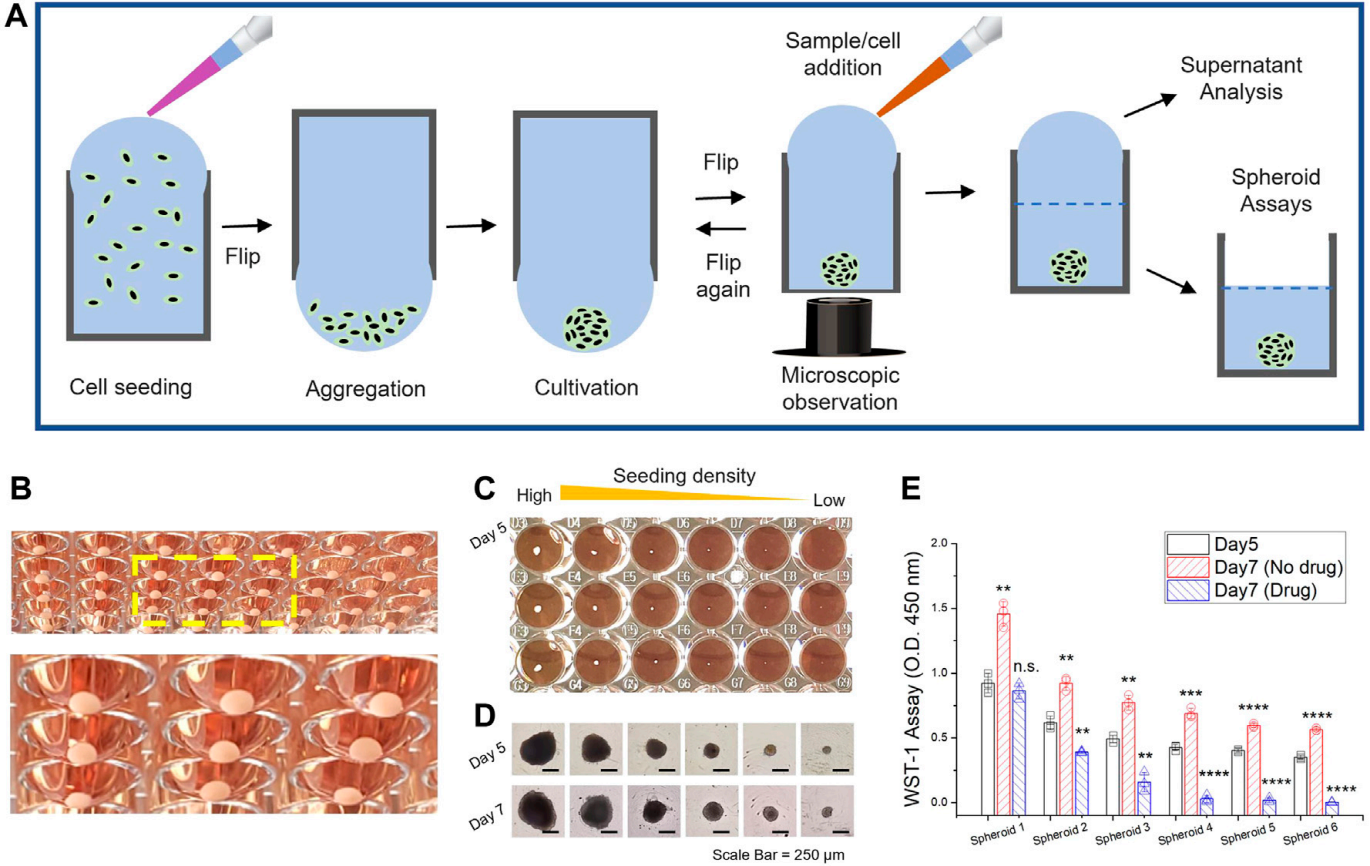
3D spheroid generation through well-plate flip. **(A)** Schematic workflow of tumor spheroid generation and sample management for endpoint analysis. **(B)** Large 3D spheroid of HCT116 cells, uniformly generated at the bottom of the flipped hanging drop in a standard 96-well plate. The 3D spheroids were cultivated for 2 weeks with manual replacement of culture media. The yellow dash box indicates a magnified view of the spheroids in hanging drop formation **(C)** 3D spheroid generation with different seeding concentrations of HCT-116 cells **(D)**. Microscope images after cultivation for 5 and 7 days. Scale bar = 250 μm. **(E)** WST-1 assay to assess 3D spheroid proliferation in Day 5 and Day 7; Nondrug treatment and drug treatment of 5-azacytidine (0.5 μM). Data represent mean ± SD (*n* = 3, biological replicates per condition, *p < 0.05; **p < 0.01; ***p < 0.001 and ****p < 0.0001 Significance by Student’s *t*-test.). The results were statistically compared with the groups (on Day 5)*.* **(C–E)** The initial seeding density of HCT116 cells was from 2 × 10^4^ (Spheroid 1) to 3 × 10^2^ (Spheroid 6) cells per well (a two-fold decrease in each titer).

Hanging drop provides a seamless dimension without spreading and has been used for various biological studies involving microbes to neural tissues over the past several decades (Harrison, 1906; Reinhoff, 1922; Kamiyama et al., 2012). For generating scaffold-free tumor models, the overfilled 96-well plate can be inverted to generate a pendant hanging drop after seeding cells in each well (Figure 2A). Cellular clusters form at the bottom center of the drop by gravity due to cell–cell interactions. In addition to the merits of generating the concave bottom in a hanging drop by gravity in a 96-well format, other experiments are possible. First, microscopic observation and addition of different compounds/cells at multiple time points are possible after spheroids temporarily settle at the bottom of the well-plate. Uneven or loose clusters could be rearranged by flipping again, which leads to improved sphericity of 3D spheroids. Additionally, the spheroidal cell culture media contain various types of molecules that can be used as indicators of abnormal cell metabolism or for further analysis. In particular, the 3D spheroid models generated by our approach enable endpoint analysis without the need for additional recovery and are compatible with standardized instrumentation that will accommodate the well-plates. These advantages are invaluable compared to 2D culture systems and other spheroid generation techniques (shown in Tables 1, 2).

**TABLE 1:** Summary of spheroid formation techniques along with advantages and disadvantages.

Methods	Advantage	Disadvantage
Conventional hanging drop	• Simple	• Low throughput
	• Inexpensive	• Long-term culture difficult
	• Uniform spheroid size	• Not efficient for media exchange
		• Small culture volume
		• Labor intensive and time consuming
		• No cell-ECM interaction
		• Transferring of spheroids for analysis
		• Not compatible with most plate readers
		• Evaporation control required
Nonadherent surface (Liquid overlay method)	• Easy to use	• Long-term culture difficult
	• Inexpensive	• No cell–ECM interaction
	• No specialized equipment	
Cell suspension culture (Rotary cell culture system)	• Simple	• No individual compartments for spheroids
	• Mass production	• Nonuniformity (size, composition)
	• Long-term culture	• High shear force
	• Good viability	• No cell–ECM interaction
		• Specialized equipment
Microfabrication (microfluidic device)	• Continuous perfusion	• Difficulty collecting cells for analysis
	• Precise handling	• High complexity
	• Flexibility to study design	• Not available to most users
		• Expensive
Magnetic levitation	• Rapid method to develop heterotypic spheroids	• Requires the magnetic nanoparticles
		• Nanoparticles’ interference to spheroid assay
		• Difficulty in scale-up
		• Limited spheroid formation
		• Not available to most users
This work (well-plate flip)	• Simple to use	• Static conditions
	• No additional cost	• Evaporation control required
	• Long-term culture	
	• High reproducibility	
	• Accessibility to any users	
	• Co-culture ability	
	• Scaffold-based culture	
	• Noncontact environment	
	• Compliant with high-throughput screening	

**TABLE 2:** Comparison of 96-well-based commercialized product [Hanging drop plate and Ultra-low attachment (ULA) plate] and our well-plate flip approach.

	Commercialized products		Well-plate flip (WPF) approach (this work)
	Hanging drop plate	ULA plate	
Price (in US Market)	$ 30–60 (USD/plate)	$ 30–70 (USD/plate)	Less $1 (USD/plate)
Format	Special plate (96 well) + Hanging drop insertion	Special plate (surface coated 96 well)	Any standard 96-well plate
Working volume	10–30 μL/well	100–200 μL/well	300–1200 μL/well
Long-term culture	Up to 6–7 days	Up to 1–2 weeks	Over 1 month
Media replacement	Difficult	Amenable	Amenable
Microscope observation	Difficult	Amenable	Amenable
Scaffold-based Culture	Difficult	Hard to maintain	Amenable

The organization of cellular clusters results from anchorage-independent growth in which geometrical features influence the spheroid morphology. A contact-free environment in hanging drops achieves a high level of consistency and reproducibility without any possibility of surface adhesion (Figure 2B). The controlled number of initial cells seeded in a well can be correlated with the size of the spheroidal clusters (Figures 2C,D, Supplementary Figure S2). The results of the spheroid proliferation assay with drug treatment (Figure 2E) and extracellular ATP level (Supplementary Figure S3) in the culture supernatant were demonstrated by endpoint analysis in a 96-well format. Universal 96-well platforms enable a manual exchange of the culture media by pipetting the liquid manually or through semi-automated instruments such as the liquid handler. A well to well transfer technique through liquid contact can be utilized not only to avoid shear damage but also to renew the growth milieu (Supplementary Figure S4). Thus, the WPF approach provides stable culture conditions for long-term culturing with a large working volume of media in each well that can be used for downstream experiments. That said, downstream analysis of tumor heterogeneity or modulation/alteration of gene expression profiles, quiescence, and necrosis (Cui et al., 2017) is beyond the aim of this study.

### Long-Term Spheroid Cultivation

**FIGURE 3:**
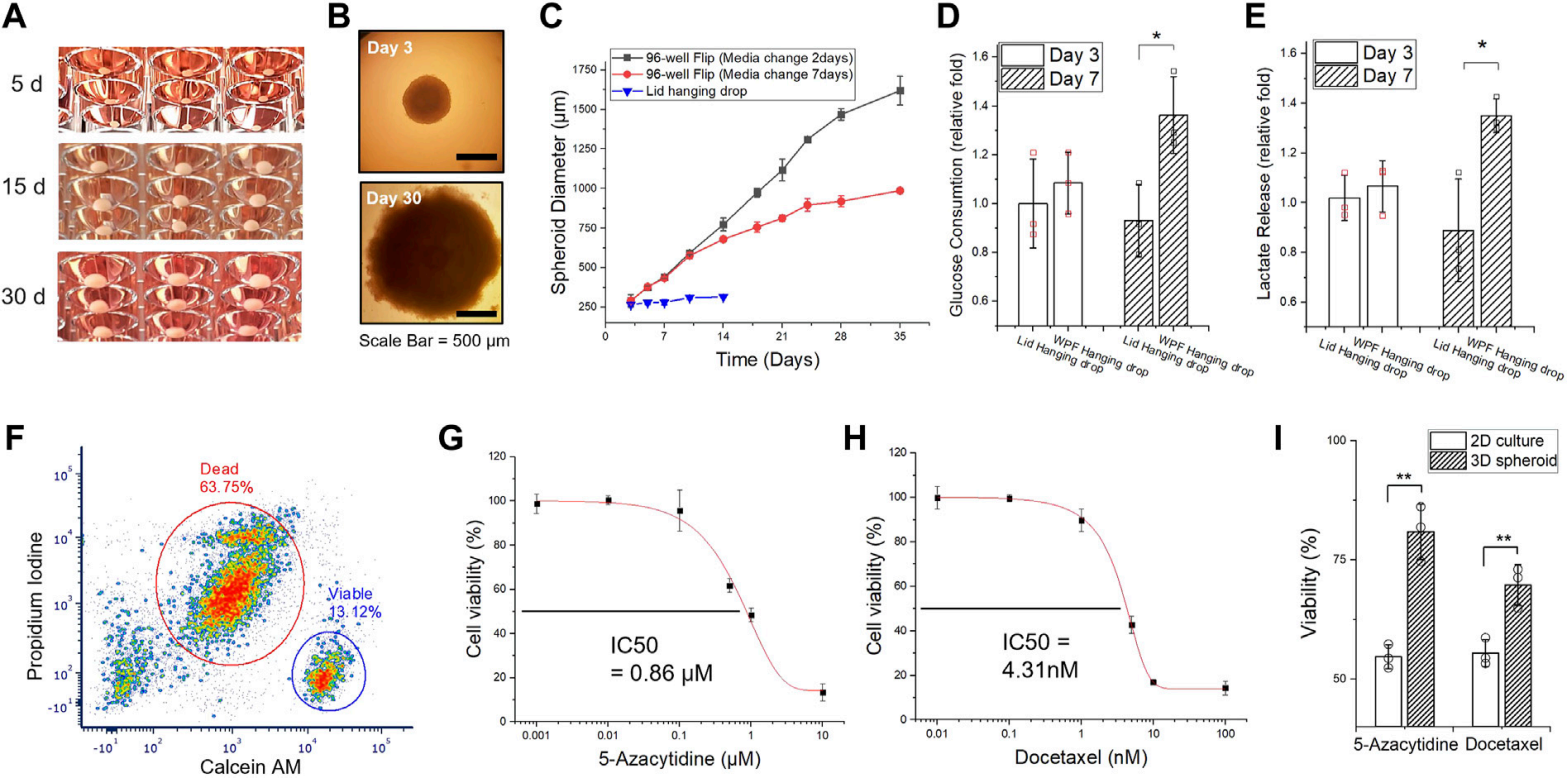
Long-term culture of 3D HCT116 spheroids. **(A)** Images of 3D spheroids cultivated for an extended time (Day 5, Day 15, and Day 30). **(B)** Microscopic images of 3D spheroids (on Day 3 and Day 30). **(C)** The size measurement of HCT116 spheroid for 35 days. The medium was manually replaced after Day 7 (black line: Every 2 days, red line: Every 7 days) through a well-well transfer technique (Supplementary Figure S3). **(D)** Glucose uptake and **(E)** lactate release of 3D spheroids on Day 3 and Day 7, generated in different conditions, flipped well-plate method and conventional lid hanging drop method. **(F)** Flow-cytometric measurement of HTC116 tumor spheroids with a 1-month culture. The spheroids were dissociated completely and stained with Calcein-AM and propidium iodide (PI) for live/dead assay. **(G)** 2D cell death index determination of 5-azacytidine and **(H)** Docetaxel. The half the maximal inhibitory concentration (IC50) values of the drugs were determined from the results of the 2D responses of HCT116 cells. **(I)** Discrepancy of cell viability assay between 2D and 3D cultures with IC_50_ of 5-azacytidine and docetaxel. All data represent mean ± SD (*n* = 3, biological replicates per condition); *p < 0.05; **p < 0.01; Significance by Student’s *t*-test.

First, we intentionally examined 3D spheroids cultivated for over 1 month by manual replenishment of culture media (Figure 3A, Supplementary Figure S5). In Figure 3B, the form of a 3D HCT116 spheroid manifests the feature of solid *in vivo* tumors with a distinct proliferating periphery, quiescent layers, and necrotic core (Costa et al., 2016; Nath and Devi, 2016). Under *in vitro* conditions, an empirical model of avascular 3D spheroids formulated by the Gompertz function of growth (Costa et al., 2016; Han et al., 2021) was shown to reach a growth plateau under depleted nutrient conditions within a few days (<5–7 days) of growth (Foty, 2011; Tung et al., 2011; Zhao et al., 2019). Experiments show that with appropriate culture conditions and media replenishment, HCT116 spheroids could be grown in a contact-free environment for up to 1.5 mm diameter, reaching a growth plateau in 1 month, as shown in our study (Figure 3C). Glucose consumption and lactate production are correlated with spheroid volume and nutritional states as well as the growth of proliferating regions. In Figures 3D,E, spheroid glucose uptake and lactate release rates were higher compared to spheroids by conventional methods on the lid of a plastic plate. The association of spheroid size accounted for the rates of nutrient consumption and production as the spheroids grew. The observed increase in glucose consumption and lactate production with growth in spheroids is mainly due to the high accumulation of cells in a proliferating and quiescent state. The presence of central necrosis core might have no effect on the rates of nutrient uptake (Dini et al., 2016). Consequently, among many factors contributing to culture conditions, nutritional considerations are a primary determinant of the size of avascular tumor spheroids (Conger and Ziskin, 1983). We expect that the avascular tumor models *in vitro* could be cultured continuously for a prolonged time period with enough nutrients.

The flow cytometry results of the live/dead assay revealed the portion of live and dead cells associated with structural heterogeneity. The HCT116 spheroid cultivated for 1 month exhibited over 60% cell death, while the HCT116 spheroids cultivated only for a short period (∼5 days) showed that most cells are still alive (Figure 3F, Supplementary Figure S6). The results imply that *in vitro* tumor models, even in isogenic tumor cells, require an extended culture time to develop quiescent layers and necrotic core to sufficiently represent tumor structural heterogeneity in a 3D milieu. Further, the biological relevance and the inconstancies of the micron-sized spheroids (<200–300 μm in diameter) developed within 1 week or less (Mehta et al., 2012; Choi et al., 2014) have been questioned because a necrotic phenotype is a typical feature of tumoral volume growing *in vivo* or *in vitro* (Dini et al., 2016; Lee S. Y et al., 2018). 3D spheroid models should encompass this characteristic in the center of spheroids by up-/down-regulating appropriate genes and/or pathways (Berghe et al., 2000; Sever and Brugge, 2015). As heterogeneity is an intrinsic feature of tumor models, the WPF system can be used as a reproducible tool for understanding tumor heterogeneity, depending on size-varied tumor models cultivated for an extended time.

Solid tumor-like properties of 3D spheroids and their resistance to therapeutics have been reported in the past studies (Gencoglu et al., 2018; Aleksakhina et al., 2019). We determined the half maximal inhibitory concentration (IC50) of two anticancer drugs, 5-Azacytidine and docetaxel, in a comparative study between 2D and 3D cultures (Figures 3G,H). In Figure 3I, a drug–response evaluation between 2D and 3D culture models shows the resistance of 3D spheroid models. Past studies also reveal inconsistent results due to a variety of intratumor and environmental factors. Most malignant cells exhibit anchorage-independent growth and tend to self-aggregate (Supplementary Figure S7). Nevertheless, the compactness and cohesive force of such spheroid formation considerably vary with the type of malignant cells, resulting in different expression levels of membrane proteins (*e.g.*, integrin, cadherin) and extracellular matrix proteins (Białkowska et al., 2020). Further, not all types of malignant cells could form dense cellular aggregates (Han et al., 2021) because even a minor physical perturbation may disrupt the 3D cellular cluster aggregates. Substantial differences in gene expression profiles, morphological phenotype, proliferation capacities, surface epitope expression, biological markers, metabolism, and metastatic potential (Ferretti et al., 2007; Fares et al., 2020) have also been noted.

### *In situ* Tumor Spheroid Assays

Tumor spheroids can settle at the bottom of the well-plate and be used for subsequent operations at different periods, where the only procedure required is flipping of the 96-well plate compatible assays. Congruent to the concept of *in situ* 96-well-plate-based spheroid development, we further demonstrate the suitability of the WPF method by evaluation of spheroids without recovery: cellular motility, matrix embedment, co-culture, and dissemination.

**FIGURE 4:**
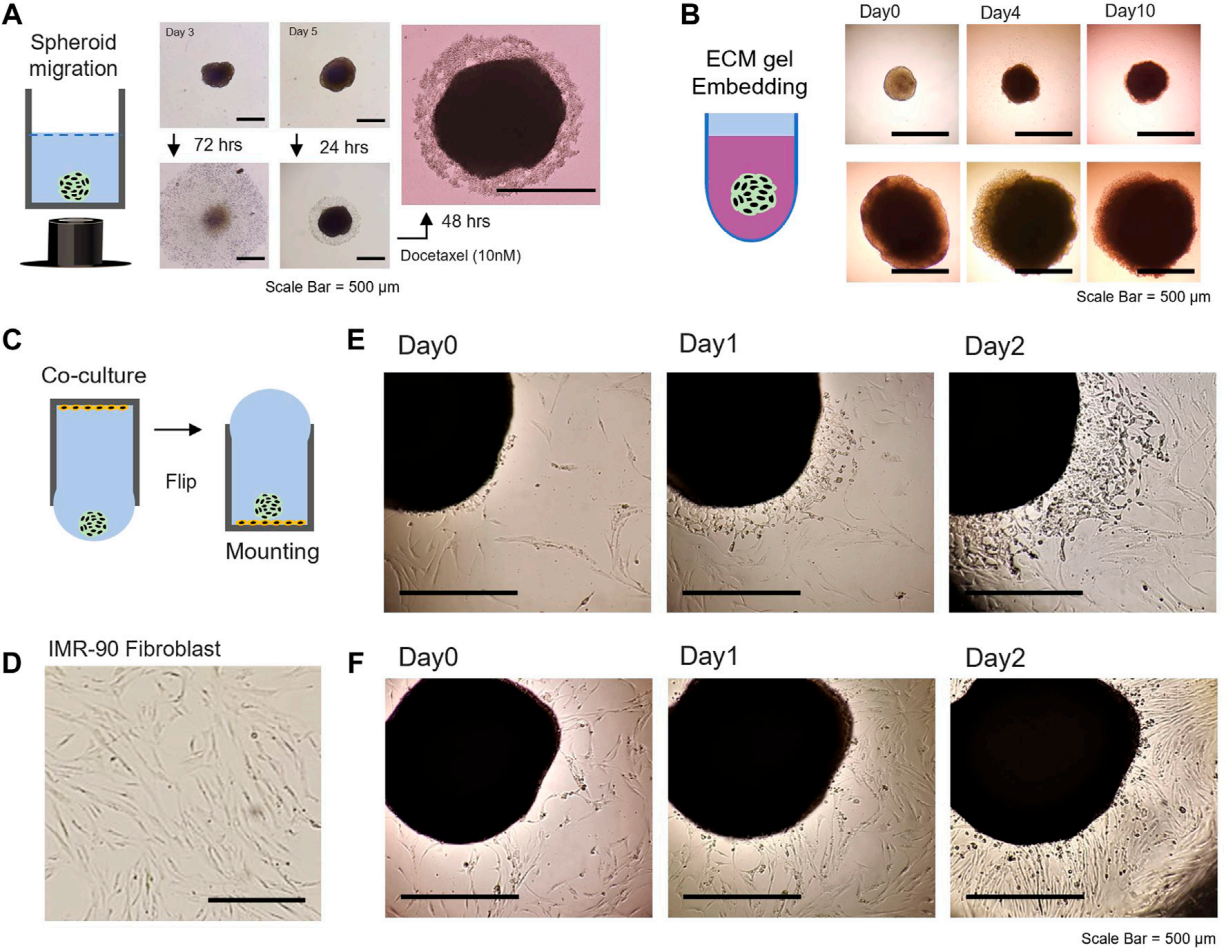
Evaluation of 3D spheroids; cellular motility, matrix embedment, co-culture, and dissemination. **(A)** Tumor spheroid migration on the 2D bottom of 96-well plate after flipping [middle panel: for 72 h, right panel: drug treatment of docetaxel (10 nM) after 24 and 48 h]. **(B)** Tumor spheroid embedding assay in the environment of ECM-like hydrogel. 3D HTC 116 spheroids with different sizes (cultivated for 5 days, upper panel and 15 days, lower panel) were embedded in Matrigel and monitored for up to 10 days. **(C)** Schematic illustration of a WPF approach for co-culture studies. **(D)** Image of the morphology of IMR-90 fibroblasts adhered to the bottom of a 96-well plate. 3D spheroid dissemination on the 2D substrate with IMR-90 lung fibroblast. **(E)** Microscopic image of 20% confluency and **(F)** 50% confluency of IMR-90 fibroblasts with 3D HCT116 spheroid (prepared for 10 days). The scale bar is 500 mm.

It is known that cell migration in many biological processes, including cancer progression, is affected by various chemical and physical processes. Figure 4A shows that the tumor spheroid can be dispersed on a 2D plate, assumed to be the relocation of many cells in the division from the free edge of cells to the top of the cluster periphery. The central region of the spheroid is distinct, as the spheroid halted its spread on the 2D plate due to drug treatment. The inhibition of migratory patterns associated with the formation and growth of metastatic carcinoma cells can be utilized to unveil a distinct feature among different carcinoma cell lines. Further, we examined 3D hydrogel embedded assays, by which the tumor spheroid was embedded in ECM gel derived from basement membrane extracts (Figure 4B). Many previous studies have reported invasive and sprouting capabilities of 3D tumor spheroids embedded in ECM-like gel over time, with a mix of ECM proteins and growth factors (Vinci et al., 2012; Vinci et al., 2015) (Supplementary Figure S8). HCT116 spheroids appeared to be condensed for 2 days when embedded in the ECM-like gel with a lack of certain factors that can induce directional cell migration or proliferation (Supplementary Figure S9). This is at least partly due to HTC116 spheroids exhibiting compact and dense solid structures in the microenvironment. The self-assembled isogenic spheroid embedded in ECM-like gel might display an intermediate-complexity between 2D monolayer cell populations and *in vivo* solid, dense tumors (Han et al., 2021). Thereafter, as growth continued, slow migration was observed in all directions with a distinct proliferation at the periphery for the next 7 days. The results indicated that the proliferative region would become dense with the physical constraint of the gel matrix, involving complex homophilic interactions of cell–cell adhesion observed in solid tumor development.

In the tumor microenvironment, metastatic cancer cells and stromal cells interact with an intricate cross-talk within tissue microenvironments associated with cell motility during tumor progression (Bussard et al., 2016). The WPF method was utilized in co-culture studies of 2D stromal cells with spheroids and subsequent dissemination assay (Figure 4C). Microscopic observation shows the spread of 3D spheroid on a 2D planar substrate with IMR-90 lung fibroblast (Figure 4D). The cell confluency of the IMR-90 fibroblast on the substrate greatly influenced the migratory characteristic of the spheroid on the 2D substrate (Figures 4E,F). Further, monitoring two types of spheroid models on stromal cells might be possible by transfer of spheroids generated separately under controlled-experiment conditions to observe migratory patterns and/or interactions with each other. Past efforts have characterized heterogenetic features of various types of 3D spheroids. However, numerous biological factors related to proliferation, migration, and invasion, which are important hallmarks of cancer metastasis (Gao et al., 2005; Fares et al., 2020), have remained elusive. Our conceived approach in a 96-well hanging drop with adequate nutrients could generate uniform and scalable 3D spheroids (>1.5 mm in diameter) that can be cultivated for over 35 days and possibly for up to 2 months.

### Scaffold-Free/Scaffold-Based 3D Culture

**FIGURE 5:**
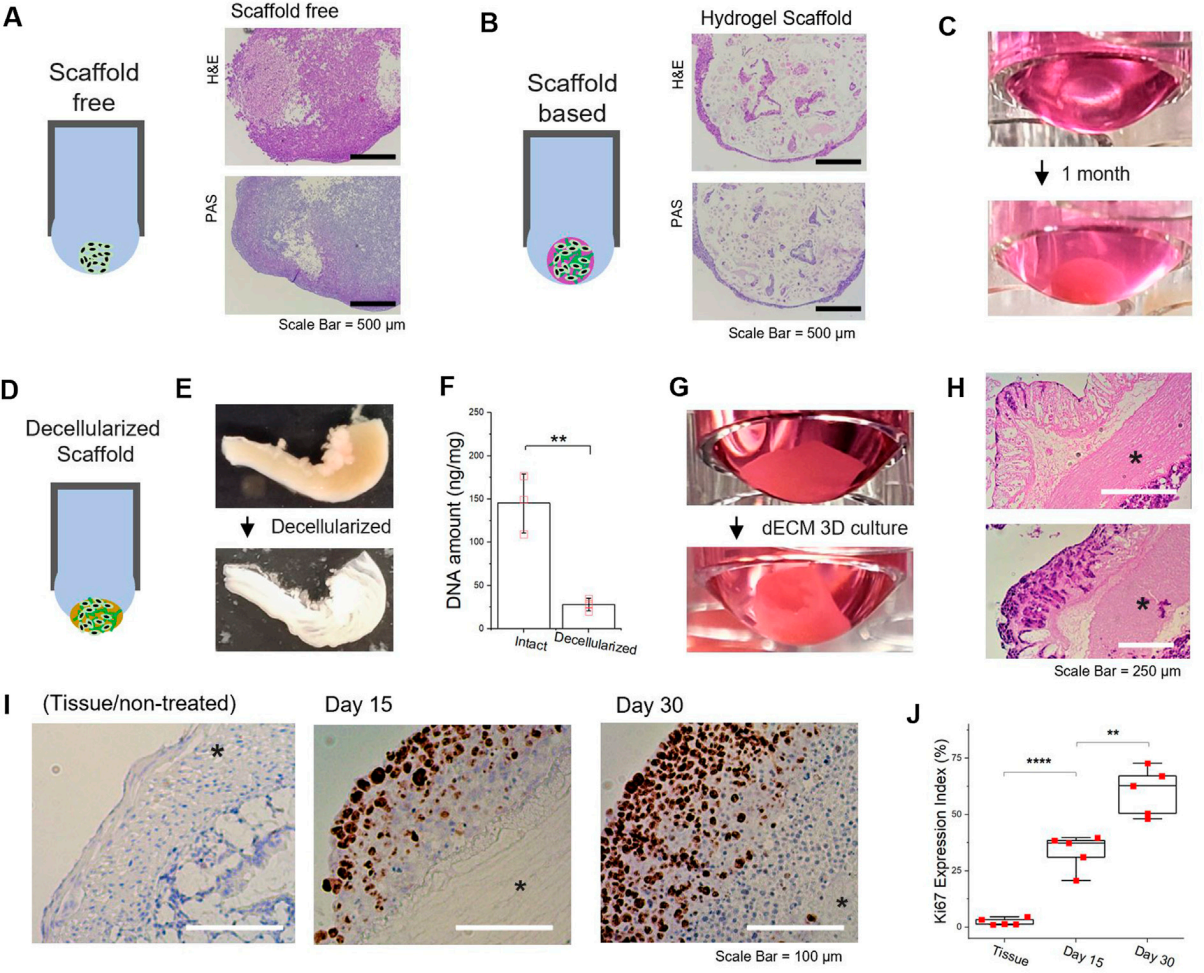
Scaffold-free/scaffold-based 3D culture of tumor spheroid generated in hanging drop formation through well-plate flip. (**A,B)** Comparison of scaffold-free/scaffold-based 3D spheroid and representative image of Hematoxylin and eosin (H&E) and periodic acid–Schiff (PAS) staining of scaffold-free/scaffold-based 3D HCT116 spheroid. A mixed gel of alginate (0.5 % wt) and gelatin (1 % wt) was utilized as a 3D substrate. **(C)** Images of 3D cell culture based on the alginate-gelatin hydrogel scaffold for 1month in hanging drop formation. **(D)** Schematic of decellularized scaffold-based 3D tumor spheroid generated by hanging drop formation. **(E)** Comparison images of decellularized mouse intestine **(F)** DNA quantification of mouse intestine samples, pre- and postdecellularization process. **(G)** Images of 3D cell culture based on the decellularized intestine for 1 month in hanging drop formation. **(H)** Histologic H&E analyses of decellularized intestine-based 3D tumor cell culture. upper: day 15 and lower: day 30 (*muscularis externa). **(I)** Immunohistochemistry images for Ki67 and hematoxylin-stained sections. Brown nuclear stain indicates Ki67 positive (left: Intestine tissue, middle: dECM-based tumor cultured for 15 days, right: dECM-based tumor cultured for 30 days) (*muscularis externa). **(J)** Percentage of Ki67 expression index (the fraction of Ki67-positive cells) by immunohistochemistry. Data represent mean ± SD; NS, not significant; *p < 0.05, **p < 0.01, ***p < 0.001, and ****p < 0.0001 by Student’s *t*-test. **(F)** *n* = 3 per condition; **(J)** *n* = 5 per condition.

Despite the usefulness of 3D spheroidal cultures in tumor malignancy studies, a gap still exists between *in vitro* and *in vivo* models. A key difference is the absence of cell–matrix (i.e., ECM components) interactions (Li and Kumacheva, 2018), which make it biologically relevant; however, other inconsistencies exist in the 3D spheroid formation generated by other platforms. To simulate *in vivo* behavior in a 3D microenvironment, scaffold-based strategies (Rijal and Li, 2017; Li and Kumacheva, 2018) have been shown to be more amenable and biologically more meaningful. We adopted a hydrogel scaffold-based 3D culture system in a hanging drop format to demonstrate the concept. When scaffold-free and scaffold-based spheroids are grown under the same conditions for 1 month, a substantial difference was observed (histological analysis shown in Figures 5A,B). Growing cells, protruding at the surface of the hydrogel matrix, gradually covered the scaffold structures by forming a proliferating periphery (Figure 5C). Wherein, the biological activities of the tumor cells, either self-aggregated or confined in the artificial matrix, could be passively affected by the physicochemical cues with respect to their surroundings, and *vice versa* (Rijal and Li, 2017).

### Decellularized Matrix-Based 3D Culture

To further enhance the physiological relevance, we utilized decellularized tissue matrix to preserve the biophysical and biochemical cues for tumor spheroid cultivation (Hussey et al., 2017; Meran et al., 2020). By extending the scaffold-based approach, we developed natural tissue matrix-based 3D culture models that can be sustained in a hanging drop platform as an *in vitro* tumor tissue analog (Figure 5D). An acellular natural matrix, referred to as the decellularized extracellular matrix (dECM), was utilized after the removal of resident cells (Figure 5E) from the mouse intestine, where most DNA contents were eliminated (Figure 5F). It should be noted that the architectures of the intestinal tissue, including submucosa and muscular layers (*e.g.*, muscularis externa), were preserved after decellularization (Supplementary Figure S10). As HCT116 cells were re-cellularized at the intestine-derived dECM, the structural morphology of dECM-based tumor culture in hanging drop was gradually reconfigured to the original shape of tubular intestinal structures (Figure 5G). Histologic H&E analyses of the dECM 3D culture showed HCT116 cells predominately proliferated at the mucosal surface of the acellular matrix (Figure 5H). Furthermore, Ki-67 positive tumor cells could be found at the exterior regions of the intestine-dECM (Figure 5I). As time progressed, the percentage of Ki-67 positive tumor cells significantly increased (Figure 5J). The results imply that mechanical properties of the tissue of interest could be retained and the biological integrity preserved (Supplementary Figure S11). Thus, the dECM scaffold-based 3D cultures developed in contact-free hanging drops can effectively model the morphological features and physiological functions of the tumor microenvironment to enable further studies with WPF 3D spheroids.

## Discussion

The simple idea of the WPF method to generate hanging drop meniscus can also be exploited for purposes other than the 3D spheroidal cancer models. However, one of the major challenges in the maintenance of the hanging drop culture platform is the evaporation rates of culture media, which vary depending on experimental conditions. Generally, an unequal reduction in the volume of media in the multiwell plate occurs from the edge to the center of a plate in a humidified incubator over time (Walzl et al., 2012; Das et al., 2016). In conventional 96-well platforms [*e.g.*, agar overlay (Friedrich et al., 2009) and ultra-low attachment 96-well plate (Raghavan et al., 2016)], it is challenging to sustain uniform growth at ambient conditions for long term even with periodic replenishment of culture media. Precise control of evaporation is critical for long-term maintenance of 3D cultures. Hanging drops generated in open platforms are very susceptible to rapid water evaporation (Frey et al., 2014; De Groot et al., 2016). To address this concern, open and bottomless hanging drop systems in microfluidics were developed with dynamic shear and interconnected networks for fluid flow; however, evaporation of liquid in hanging drops was still a limitation. The loss of culture media must be compensated by the frequent addition of liquid or equipped with other auxiliaries (Park et al., 2020) to maintain the height of the suspended drop in the system. We addressed this challenge by utilizing a designed chamber or a spacer (Supplementary Figure S1) to compensate for evaporative loss.

Noncontact 3D environments to study effective cell–cell and cell–ECM interactions have utilized force-driven floating techniques (Shri et al., 2017; Chen et al., 2019). A representative example is the 3D floating culture systems based on iron nanoparticles (FeNPs) with the magnetic field as an external stimulus (Souza et al., 2010). It should be noted that the utilization of external stimuli to construct such configurations fundamentally affects cellular physiology and cell function and does not mimic the natural environment. In recent years, FeNPs have been shown to induce ferroptotic cell death (i.e., ferroptosis) (Dixon et al., 2012). The absence of ECM interactions in a tumor environment renders such experiments less physiologically relevant since mimicking *in vivo*-like cytoarchitecture in the floating culture systems has remained a challenge. To address these classical challenges, we adopted dECM-based approaches to demonstrate practical and feasible scaffold-based 3D cultures in a noncontact configuration at the bottom of a hanging drop. Historically, hanging drop preparation has been applied to cultivate intact tissue fragments dating back hundred years (Harrison, 1906). The concept of scaffold-based 3D culture in hanging drops is advantageous to query the response of both cell–cell and ECM–cell interactions over an extended time period under well-controlled contact-free environment. Although a huge gap in knowledge exists in the development of 3D culture models and *in vivo* tissue microenvironments, the degree of flexibility and ease of fabrication have significant potential in addressing a wide range of applications from intact tissues to multicellular organoid models in scaffold-free/scaffold-based format.

The central interest in 3D spheroidal cultures is in high-throughput screening/manufacturing and its *in vivo*-like tissue features to obtain mechanistic/endpoint information, which is very cumbersome in animal models. For example, hundreds or thousands of multiple cellular clusters could be concurrently generated and arranged in a single chip-like microfabricated device containing thousands of microwells (Gong et al., 2015; Fang et al., 2019). For the same reason, routine assays for drug-dose efficacy were shown for spheroid size-dependent drug screening or cellular responses (Fernandes et al., 2009; Markovitz-Bishitz et al., 2010; Xu et al., 2011; Pradhan et al., 2017). As emphasized above, a primary advantage of the 96-well-based platform is its accessibility and user-friendly *in situ* endpoint analysis of biological samples without recovery or its adaptability to other standardized formats. The 3D spheroidal models generated by our WPF technique can be readily evaluated with existing instrumentation available in most laboratories. Utilizing the 3D spheroid technology, we show that routine experiments performed with 2D cultures can now be performed with 3D cultures in a standard 96-well plate in parallel.

## Conclusion

In summary, we introduce a hanging drop meniscus for 3D culture fabrication utilizing the universal 96-well-plate format. We demonstrate stable 3D culture configurations and the generation of a large 3D tumor spheroid over 1.5 mm in effective diameter in universal 96-well-plate hanging drop platforms without any extra amenities. Based on this simple strategy, we show that various scaffold-based 3D culture systems can be developed. Our approach alleviates technical barriers such as long-term maintenance and a contact-free culture environment, which are major challenges in advancing the broader use of 3D culture systems. We expect our simple approach to generating 3D culture platforms could be adopted by scientists to develop a range of 3D culture models to address problems in basic and applied biosciences. Future work could focus on incorporating different cell types and cancers as well as utilizing primary cell cultures. In the long term, we expect to establish analytical protocols based on the WPF approach (i.e., subsequent biological evaluation) to interrogate tumor heterogeneity in the context of the microenvironment.

## Data Availability

The original contributions presented in the study are included in the article/Supplementary Materials, further inquiries can be directed to the corresponding author.
