# Supplementary material for: Flipped Well-Plate Hanging-Drop Technique for Growing Three-Dimensional Tumors
Source: Front Bioeng Biotechnol. 2022 Jul 4;10:898699. doi: 10.3389/fbioe.2022.898699 (PMC9289396; doi:10.3389/fbioe.2022.898699)
Supplement: Supplementary file 1 [file DataSheet1.docx]

Supplementary Material

## Supplementary Figures


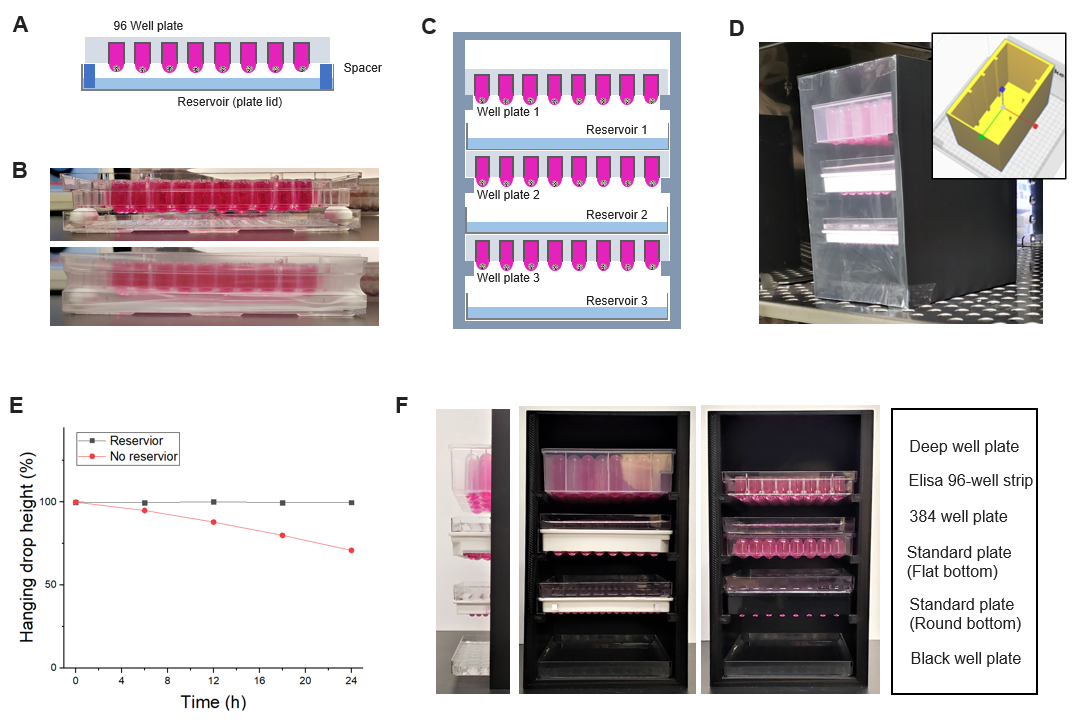


**Supplementary Figure 1. Evaporation control system in well-plate hanging drop formation. A**. Schematic depiction of a 96-well lid reservoir system with a spacer. **B**. Images of a lid reservoir system (before/after Parafilm^®^ sealing) to prevent water evaporation of hanging drops in a flipped well plate (FWP). The magnet spacer used in this figure prevents the hanging drops from contacting the surface of the lid reservoir and, then the 96-well plate was loosely wrapped with the Parafilm^®^. **C**. Schematic depiction of a proto-type chamber system to control the evaporation of multiple FWP. Multiple well-plates can be stacked in a chamber with reservoirs to compensate for the loss of evaporation. **D**. Representative image of the proto-type chamber placed in a humidified incubator that contains three different types of FWPs (inset image: 3D printer design of the chamber drawn by Sketchup software) **E**. Plot of evaporation loss of hanging drop height to compare the role of reservoirs in the chamber. The result was monitored every 6 hours in a 5% CO_2_ humified incubator at 37°C. **F**. Representative examples of multiple FWP stacked in the chamber. For experiments, 96-well products were used without any modification or treatment in this study, and all the reservoirs depicted in the schematic A and C were filled with PBS.


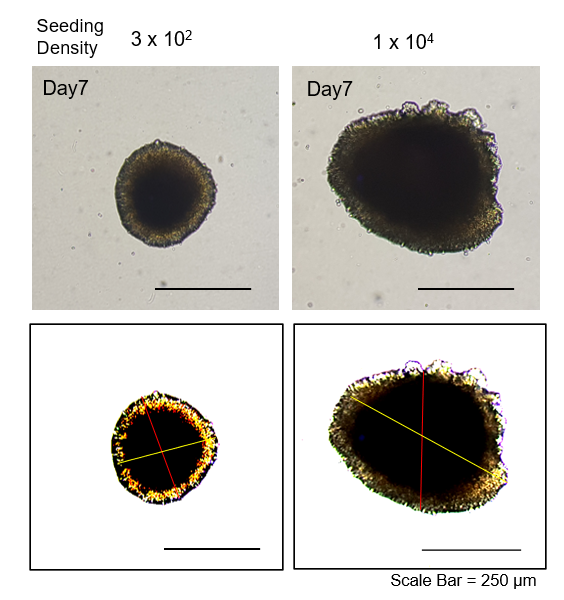


**Supplementary Figure 2. Estimation of the spheroid volume.** The volume of spheroids (V = 0.5 x Length x Width^2^) is calculated using the spheroids' major and minor axial lengths, (Chen et al., 2014) (The major axis is represented by the yellow line, which connects a single pair of the contour's longest points; the minor axis is represented by the red line, the shortest line perpendicular to the major axis.)


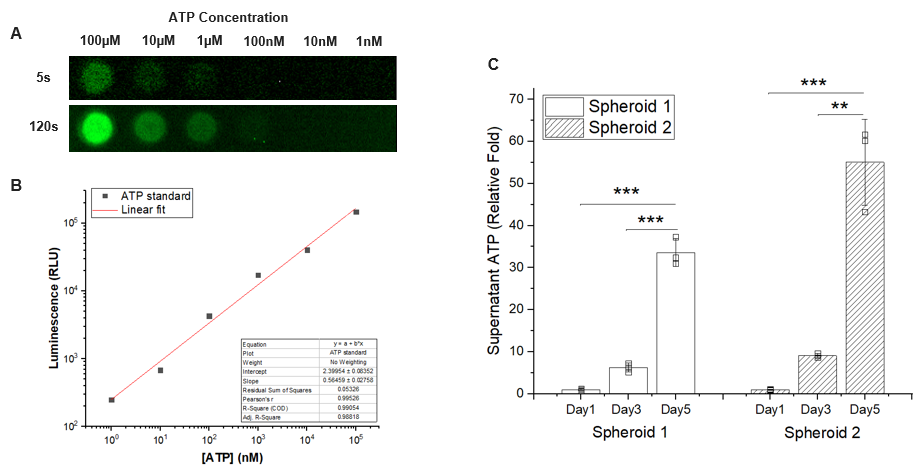


**Supplementary Figure 3. Extracellular ATP level measurement.** **A**. Bioluminescent intensity measurement. Images were acquired using a chemiluminescence gel documentation system (signal accumulation for 5s and 120s without an excitation source). **B**. ATP standard curve of bioluminescence assay. The result was obtained from a microplate reader in the bioluminescence-mode (Biotek Synergy H1) **C**. Extracellular ATP measurement of different sized spheroids. The initial seeding density of HCT116 cells was from 2 x 10^4^ cells per well (Spheroid 1: cultivation for 5 days, Spheroid 2: cultivation for 7 days in hanging drop formation). After the replenishment of culture media, ATP levels were measured after 1, 3, and 5 days by collecting the supernatant from the spheroids. The data from the bioluminescent assay represents the relative fold intensity. The data in Figure C represents mean ± standard deviation (s. d.) from three independent experiments. *, p < 0.05; **, p < 0.01; ***, p < 0.001.


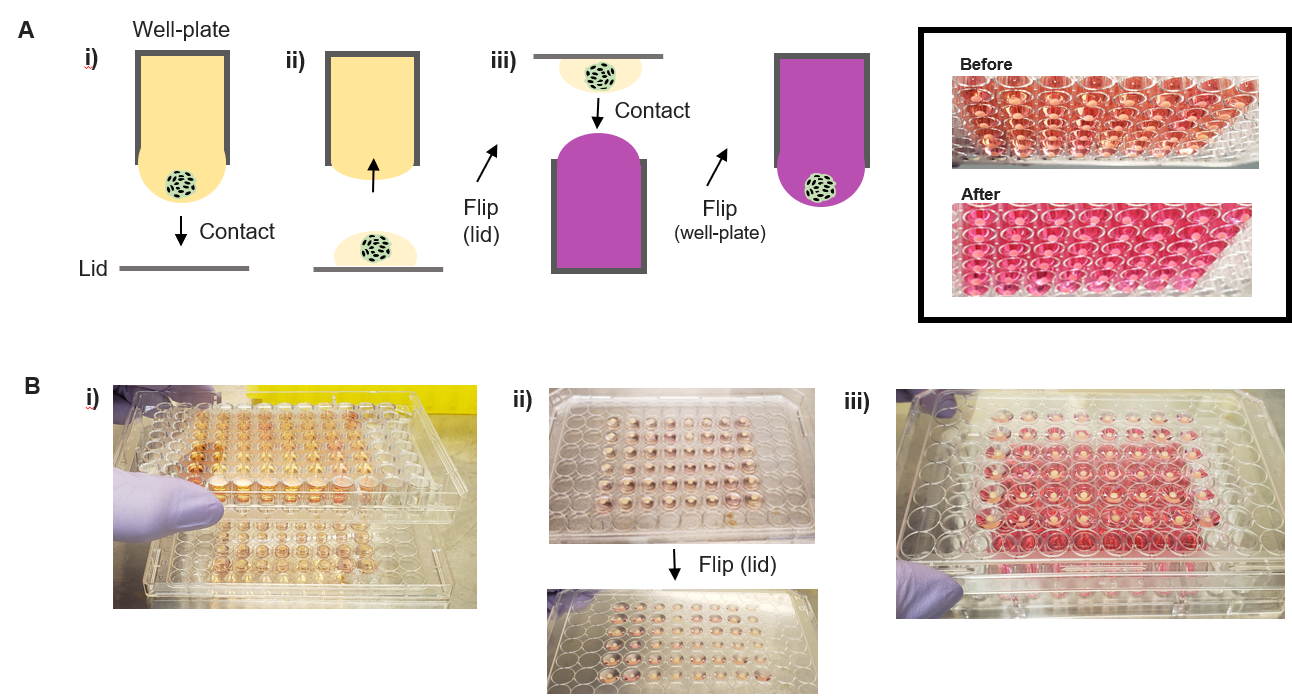


**Supplementary Figure 4. Culture media replenishment. A**. A schematic depiction of well-well transfer processes to replenish culture media. i) Once in contact, spheroidal samples were transferred onto the surface of a well-plate lid. ii) By flipping the well-plate lid again, spheroids could be suspended in hanging drops. iii) Spheroid samples were transferred from the drops to a well filled with fresh media in a 96-well plate. The 96-well plate then flip to generate a hanging drop meniscus. Comparative images of the FWP with multiple spheroids generated by hanging drop formation before/after replenishment. **B**. Images of spheroidal transfer processes responding to each step.


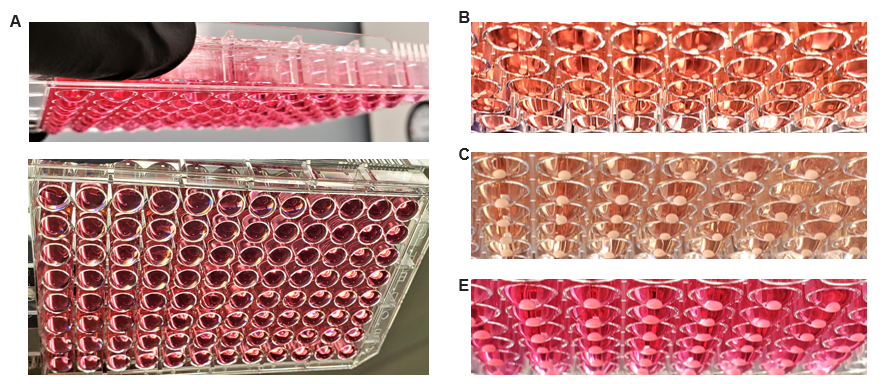


**Supplementary Figure 5. Images of flipped 96-well plate and 3D HCT116 spheroid generation. A**. A standard 96-well plate (Thermo scientific). The wells were overfilled with 440 μl volume to the maximum capacity. Bottom view (lower) of the plate on day 1 (seeding density of HCT116 cells, 500 cells per well). The spheroids were cultivated with manual media replenishment and photographed on **B**. Day 5 **C**. Day 15 **D**. Day 30.


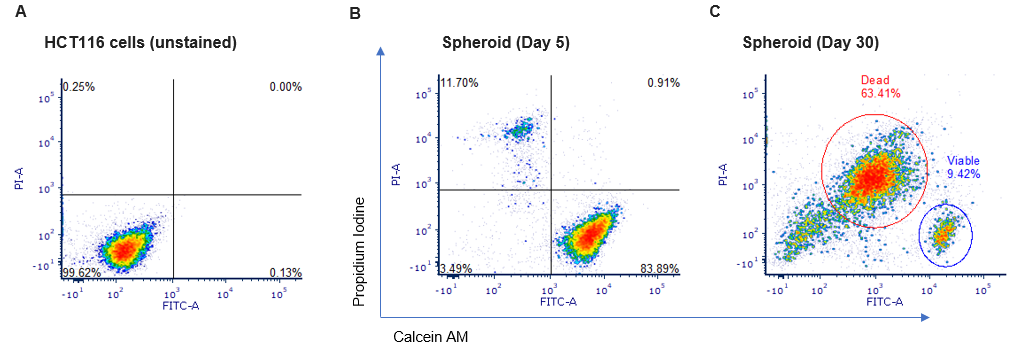


**Supplementary Figure 6. Flow cytometric analysis of 3D spheroids for Live/Dead assay. A**. Unstained HCT116 cells **B**. 3D spheroids cultivated for 5 days **C**. 3D spheroid cultivated for 30 days.


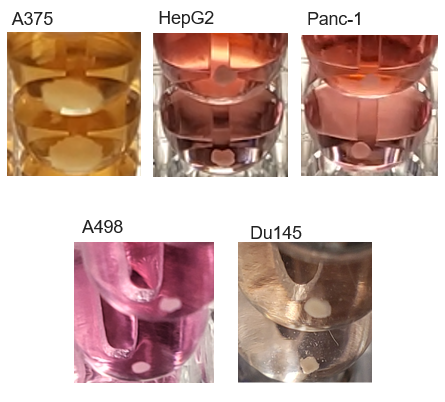


**Supplementary Figure 7. Different types of 3D tumor spheroid, generated in this study.** A375 (skin melanoma), HepG2 (hepatocellular carcinoma), Panc-1 (pancreatic carcinoma), A498 (renal carcinoma) and Du145 (prostate carcinoma).


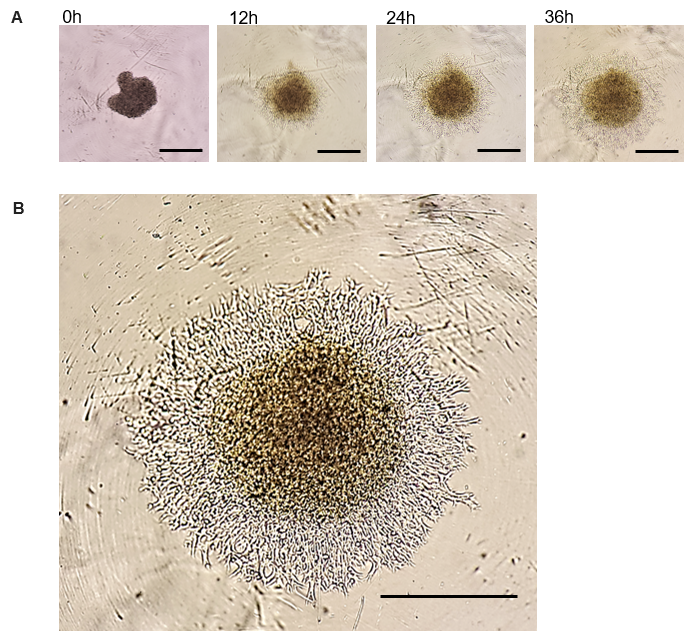


**Supplementary Figure 8. A.** Microscopic images of U87 (glioblastoma) spheroid (prepared for 3 days) sprouting in Matrigel at each time point (0, 12, 24, 36h) **B**. Magnified view of U87 spheroid after 36 hours. The scale bar is 300 μm.


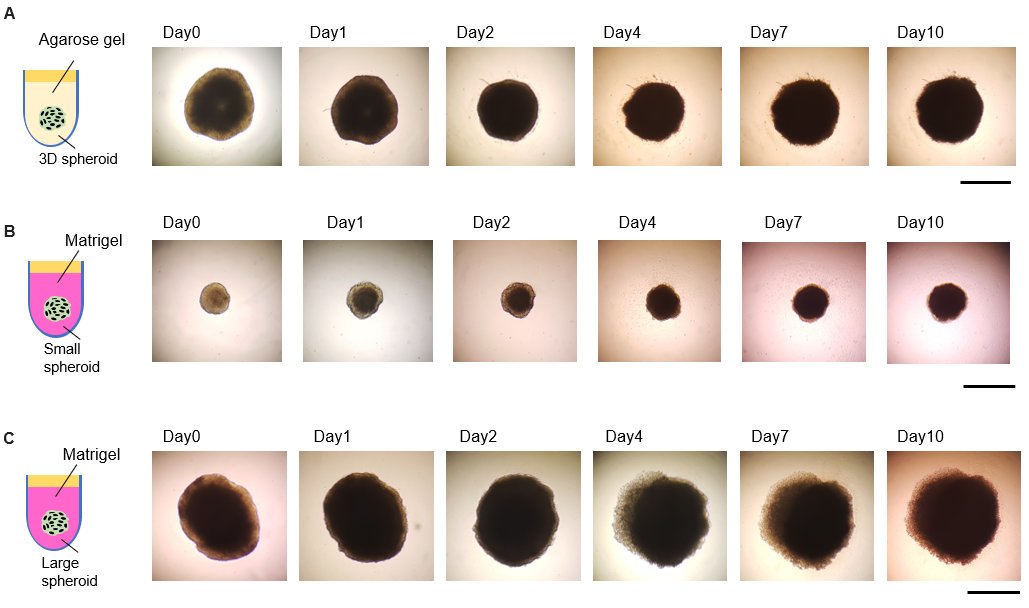


**Supplementary Figure 9. Tumor spheroid embedded in a 3D matrix gel environment.** HCT116 spheroids were embedded into **A**. Agarose gel (0.5 wt%, w/v) and **B, C**. Matrigel matrix. Large tumor spheroid (A and C; prepared for 15 days) and small spheroid (B; 5 days) were generated by the FWP method. The scale bar is 500 μm.


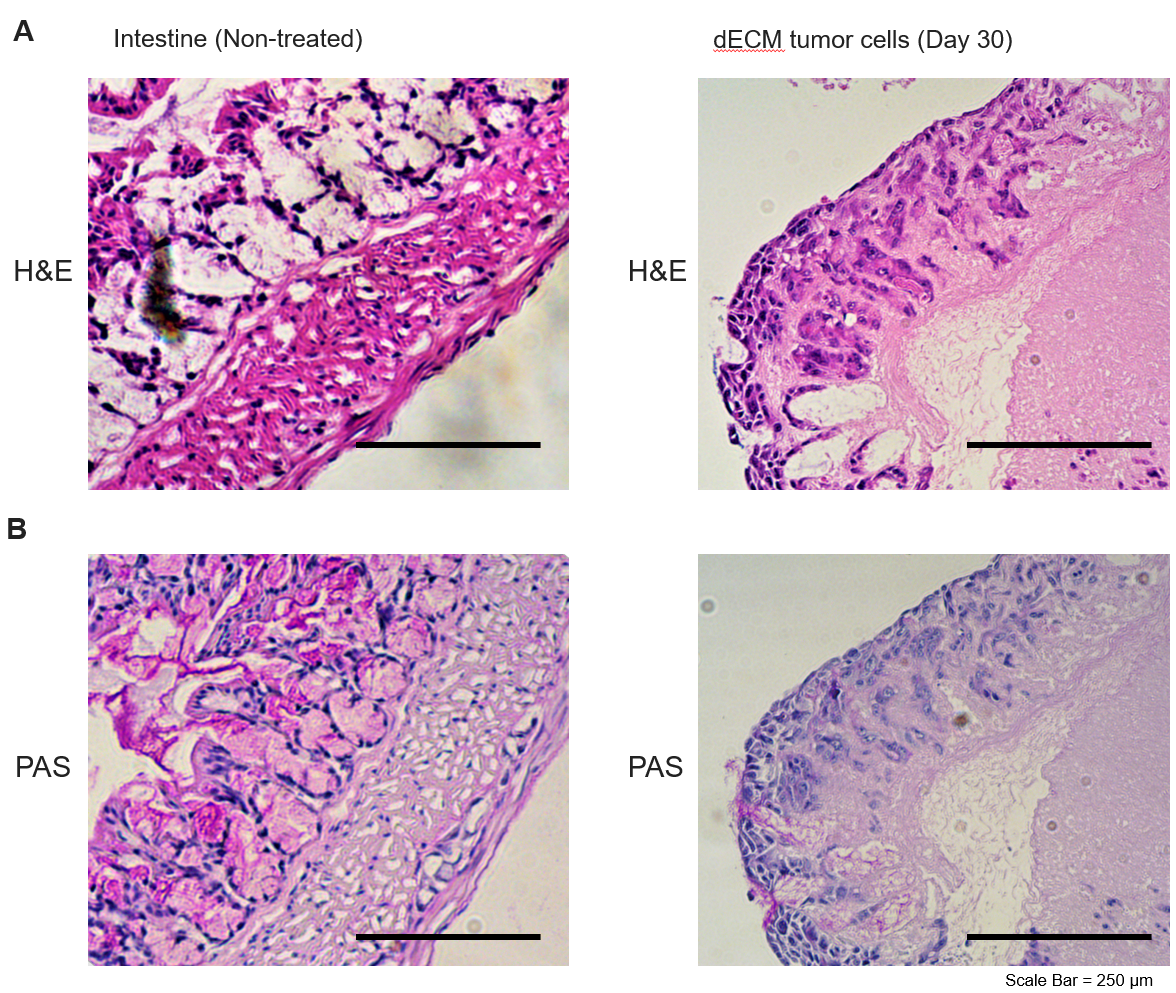


**Supplementary Figure 10.** Comparative histological analyses **A**. Hematoxylin and Eosin (H&E) staining. **B**. Periodic Acid Schiff (PAS) staining. (Left: intestine tissue before decellularization, Right: decellularized intestine-based HCT 116 cells, cultivated for 30 days). The scale bar is 250 μm.


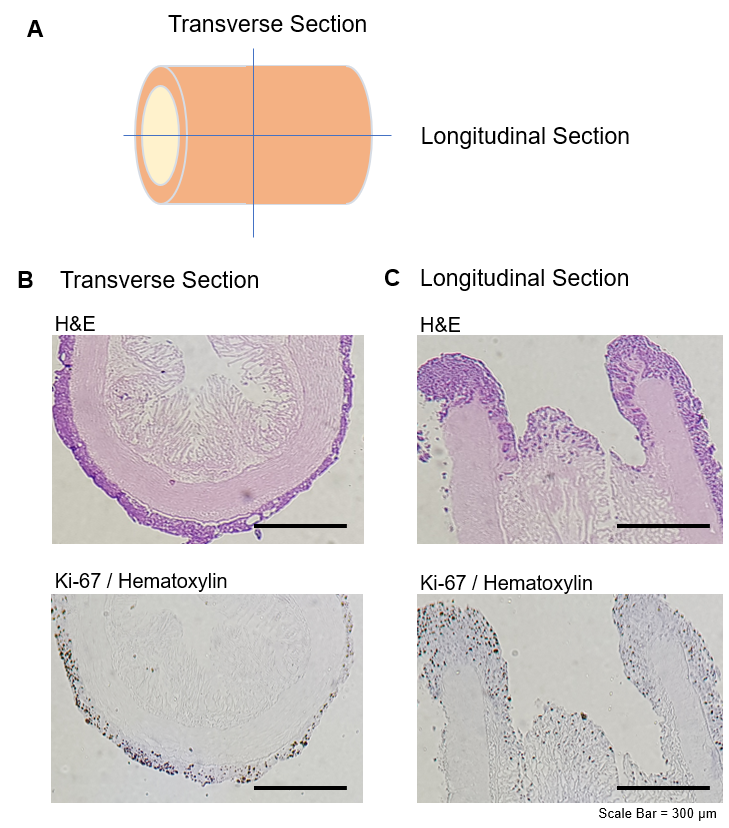


**Supplementary Figure 11. A**. A schematic depiction of the transverse and longitudinal section of the tubular intestine structure. **B**. Transverse section, and **C**. Longitudinal section of decellularized intestine-based 3D tumor cells cultured for 15 days. (B-C) Upper panel: Hematoxylin and Eosin (H&E) staining, Lower panel: Anti-Ki67 and Hematoxylin staining. The scale bar is 300 μm.

**References**

Chen, W., Wong, C., Vosburgh, E., Levine, A.J., Foran, D.J., and Xu, E.Y. (2014). High-throughput image analysis of tumor spheroids: a user-friendly software application to measure the size of spheroids automatically and accurately. *JoVE (Journal of Visualized Experiments)***,** e51639.
